# Purchasing sustainable palm oil products: narrowing the intention-behavior gap

**DOI:** 10.3389/fnut.2023.1329901

**Published:** 2024-01-04

**Authors:** Cassandra Shruti Sundaraja, Amy Dianne Lykins, Donald William Hine

**Affiliations:** ^1^School of Psychology, University of New England, Armidale, NSW, Australia; ^2^School of Psychology, Speech and Hearing, University of Canterbury, Christchurch, New Zealand

**Keywords:** sustainable palm oil, consumer behavior, intention-behavior gap, COM-B, barriers around opportunity

## Abstract

Experts on palm oil production and utilization emphasize the role of consumer purchasing power in dealing with the environmental and social impacts of the palm oil crisis -that by increasing the demand for sustainable palm oil (SPO), greater supply will follow. However, research has identified a persistent intention-behavior gap. Even knowledgeable consumers do not always follow through on their intentions to purchase SPO. Utilizing the Capability-Opportunity Motivation model of Behavior (COM-B), this article reviews important variables contributing to this intention-behavior gap. While knowledge about palm oil and SPO (capability), perceived product availability (opportunity), and pro-green consumption attitudes (motivation) are important predictors of SPO purchasing intentions, increasing these factors has been insufficient in narrowing the intention-behavior gap. Campaigns can increase knowledge about palm oil and SPO, as well as build motivation around making the ‘sustainable’ choice, but are inadequate in addressing barriers around opportunity (e.g., ease of access to SPO products). In expressing their intent to purchase SPO products, consumers may underestimate the difficulties in being able to identify these consumables (e.g., palm oil often is not clearly labeled, sustainability status may not be obvious), and locate them. In this review, we argue that while consumer behavior is important, it is insufficient to power industry-wide change toward the utilization of SPO. Greater corporate responsibility is needed to increase use of SPO in products, and make consumables containing SPO more available, identifiable, and affordable for consumers. We also suggest that national procurement policies for SPO are likely to produce longer-lasting change.

## Introduction

Palm oil is a popular, widely-used agricultural commodity that is replete with controversy. On the one hand, this relatively inexpensive oil with high yields has greatly assisted rural farmers battle poverty in producer countries of Indonesia and Malaysia (1–3). However, its growing international demand has necessitated more cleared land for plantations, which has driven widespread tropical deforestation linked to peat degradation, biodiversity loss, species extinction, and forest fires (1, 4–6). The rising global demand for palm oil is fuelled by increased *per capita* incomes, urbanization, growing consumerism, and changing lifestyles (increase in vegetable oil consumption via convenience, processed, and “junk” food; (7, 8)), and is unlikely to slow (9, 10).

### Consumers and sustainable palm oil (SPO)

The idea of ‘responsible consumption’ in the context of environmental concerns was first postulated in 1973 (11) and has since taken root, with concepts such as “green consumption,” “ethical consumption,” and “sustainable consumption” used interchangeably. This increasing interest in the role and responsibility of consumer behavior on pro-environmental outcomes has been supported by large bodies of research examining barriers and drivers to green consumption within households (12, 13). With respect to palm oil, campaigns have proposed two primary courses of action for consumers – either boycott palm oil altogether, or ensure that one’s purchases contain sustainable palm oil (SPO), although the latter is favored for several reasons. Boycotting palm oil would not only have an adverse effect on rural farmers and the economies of developing producer countries, but would also likely just shift crop-related deforestation to another oil with lower yields (1, 4, 14, 15). Most experts agree that promoting SPO is the way forward (16, 17), specifically by consumers placing pressure on manufacturers to source SPO for their products, among other suggestions like investing in companies that exclusively source SPO and purchasing only SPO products (16). However, when consumers were surveyed, they were least likely to engage in writing to manufacturers, and instead indicated that they would be more likely to avoid products containing palm oil (18, 19), and/or purchase products containing SPO (16). When educated about the importance of purchasing SPO (as versus a complete boycott of palm oil), consumers expressed their intent to make the switch, but this was not reflected in follow-up behavior (20). Therefore, the aim of this targeted mini-review is to explore this intention-behavior gap with respect to purchasing SPO products. Specifically, we aim to provide an understanding of the barriers that consumers face (which could drive this intention-behavior gap) and use this information to offer recommendations for increasing SPO consumption. Research papers and conference proceedings referencing palm oil and/or sustainable palm oil were reviewed, with a specific focus on those that target consumers. As research in this area is limited, other research into ‘green’, ‘sustainable’ and ‘ethical’ consumption were also reviewed and included when relevant.

## Drivers and barriers to purchasing SPO

**Figure 1:**
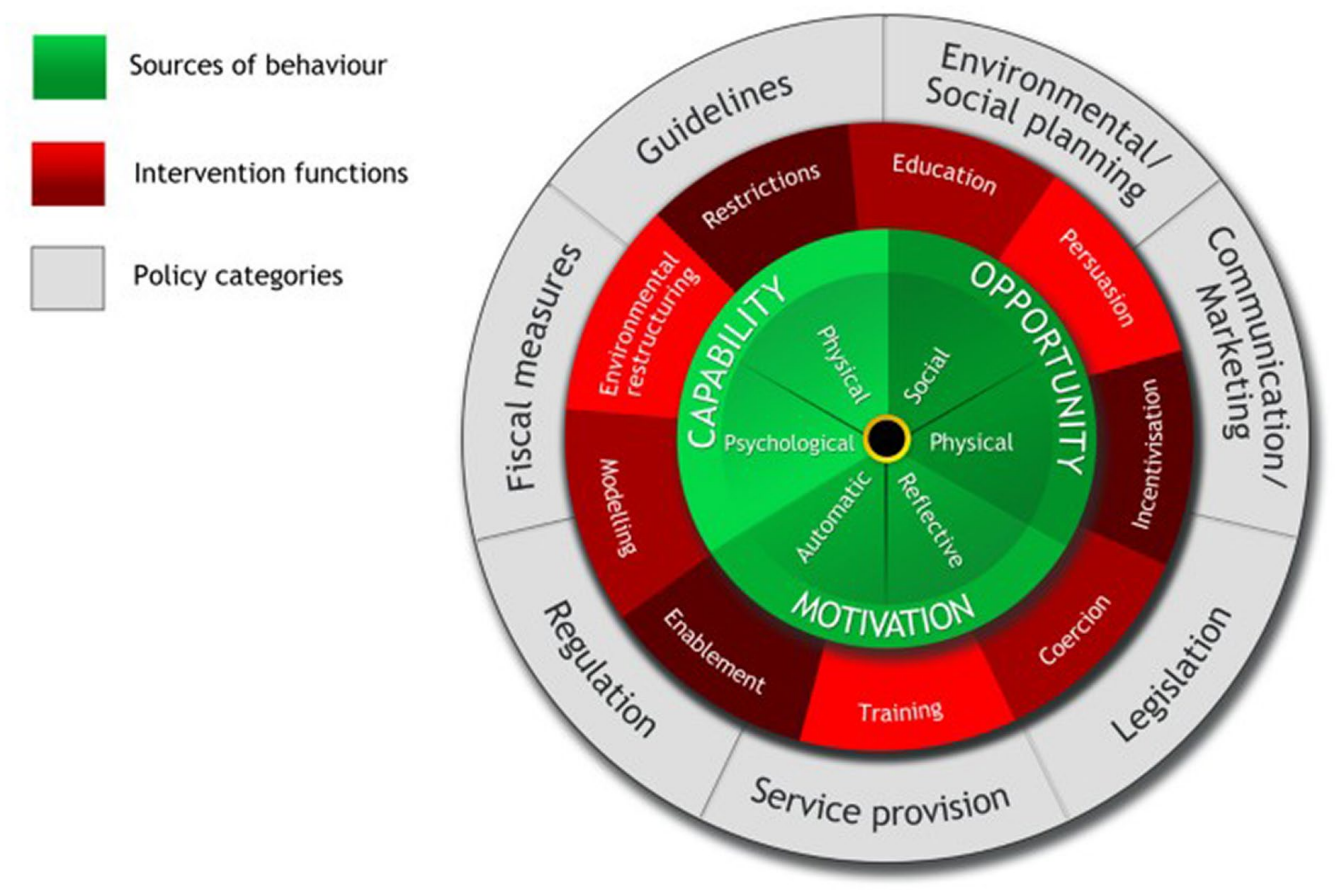
The behavior change wheel. Reprinted from “The behavior change wheel: A new method for characterizing and designing behavior change interventions,” by S. Michie, M. M. van Stralen and R. West, 2011, Implementation Science, 6 (23), p. 7. Copyright [2011] by Michie et al., licensee BioMed Central Ltd. Used under Creative Commons Attribution License: http://creativecommons.org/licenses/by/2.0.

An intention-behavior gap can be defined as the “gap between the possession of environmental knowledge and environmental awareness, and displaying pro-environmental behavior” (21), p.240. Various internal and external factors play a role in impacting green or ethical consumption (22). To better understand these factors, we will organize them within an overarching framework – Michie et al.’s behavior change wheel (Figure 1; (24)), as it allows for a comprehensive system to organize causes of behavior, while simultaneously identifying corresponding interventions and/or policy initiatives required to increase the target behavior. The inner circle of the wheel consists of Capability-Opportunity-Motivation factors (COM-B) that predict behavior directly, or in interaction with one another.

It is reasonable to expect that there could be barriers across capability, opportunity, and motivation that might explain the intention-behavior gap in SPO purchasing. A list of possible barriers and drivers that influenced consumer decision-making on purchasing SPO-containing products emerged from an in-depth qualitative study with 13 Australian participants (predominantly female) whose ages ranged from 24 to 73 with a median age of 30 (25). These findings have been organized according to COM-B and are summarized in Table 1.

**Table 1:** COM-B factors influencing the purchase of SPO (26).

Capability	Opportunity	Motivation
Knowledge about the issue	SPO product availability	Guilt
Critical thinking	Legible, clear product labels	Perceived consumer efficacy
Affordability	Product visibility	Shopping habits
Time	Social norms	Values/Moral compass
Energy		Empathy, compassion and love for the environment
		Health benefits

A subsequent follow-up to that study (*N* = 781 Australians) revealed three key predictors of SPO purchasing behavior that mapped onto COM-B: (1) knowledge and awareness of issues related to palm oil and SPO (capability); (2) believing that SPO products were easily available, termed as perceived product availability (opportunity); and (3) having pro-“green” consumption attitudes (motivation) (26). Out of these predictors, self-reported knowledge (on the environmental impacts of unsustainable palm oil, consumables containing palm oil, and the option of SPO) accounted for around 18% of the unique variance in existing SPO purchasing behavior, while perceived product availability and pro-green consumption attitudes accounted for 2 and 1% of its unique variance, respectively (26). An online intervention was designed, utilizing the behavior change wheel to guide intervention strategies based on observed barriers. The intervention specifically aimed to increase knowledge and awareness with various methods of information delivery, enhance confidence in being able to identify SPO products, and heighten pro-green consumption attitudes (20). It was administered to 628 Australians, who identified as the primary/co-equal grocery shoppers in their households (20). Participants randomly assigned to the intervention, relative to those assigned to the control group, reported significantly stronger intentions to purchase SPO products. However, importantly, a two-week follow-up indicated that almost 40% of the sample exhibited a large intention-behavior gap – they expressed high intentions, but low rates of self-reported SPO purchasing behavior (measured as the frequency of checking product labels for SPO, intentionally purchasing products containing SPO and avoiding products that contained palm oil not labeled as sustainable; (20)).

### Drivers and barriers of motivation

Research has indicated that consumers are motivated to purchase sustainably when it comes to palm oil (25, 26). Guilt and pride have been found to influence green purchasing behavior by increasing self-efficacy (27), and self-efficacy in turn was found to be more powerful than personal norms in predicting green purchasing intentions (28). A meta-analysis revealed that fear messages, when combined with high self-efficacy messages, led to the greatest increases in pro-environmental behavior (29). Further, empathy toward nature has been found to be enhanced using anthropomorphic animal imaging in campaigns and advertising, which in turn increases purchasing of green products (30). The online intervention study mentioned above (20) targeted motivation by building empathy for the declining wildlife in Southeast Asia in presenting the information on palm oil and SPO from the perspective of a baby orangutan and a Sumatran tiger cub. Additionally, it aimed to empower consumers by pairing this with information on what they could do (i.e., self-efficacy), by providing an Australian shopping guide (31) and a list of brands that consumers could purchase from (32), but this was still unsuccessful in narrowing the intention-behavior gap (20). Research in green consumption has emphasized that interventions targeting motivation alone might be less effective than addressing capability- and opportunity-related barriers (28, 33–35).

### Drivers and barriers of capability

Knowledge has been identified as an important capability-related driver of green consumption (12), and specifically SPO consumption (36, 37). Research conducted among consumers in Germany, Italy, and Switzerland revealed a negative attitude toward palm oil in general, and that most participants knew very little about SPO, (19, 37, 38). As the nature of the palm oil crisis is complex, the simplest perceived solution of avoiding palm oil altogether (16, 18) is not actually the optimal one. To understand why SPO is better, consumers would require specialized knowledge about the causes of the environmental destruction associated with palm oil, as well as the trade-offs associated with potential solutions (39). A Swedish ethnographic study stressed that consumers have limited information about the complexities of palm oil supply chains, and that there are seemingly competing environmental and socio-economic concerns, which can overwhelm decision-making (40). There are a few educational interventions that have aimed to address this lack of knowledge. Two independent interventions conducted by zoos in the United Kingdom aimed to impart information about palm oil and the importance of sustainable purchasing (41, 42). One targeted zoo visitors in general and was delivered by rangers (42), while the other conducted outreach workshops for children in school, aged 7 to 11 (41). Both these studies reported an increase in knowledge and intentions to purchase SPO, but were limited in their capacity to assess for actual behavior (41, 42). Further, the research that targeted visitors to the zoo found that engagement with rangers was low (only a quarter of zoo visitors interacted with a ranger), which translated into low impact (42), and the one that targeted school children did so with the aim of influencing the younger generation (41), but it is unclear how much of an impact these children may have had in current household grocery shopping decisions. There are two other interventional studies in this space, both conducted in Australia, that assessed immediate and follow-up self-reported behavior (20, 34). One of these approached palm oil from the perspective of orangutan conservation and was conducted with university students (34). The researchers screened a documentary (GREEN), that focused on the last days of a dying orangutan (23), and also presented facts on orangutan habitat, behavior, and intelligence, as well as how to help the conservation effort—one of these being responsible palm oil purchasing (34). The other study is the online intervention based on the behavior change wheel that targeted consumers and explained the environmental and socio-economic complexities of the palm oil issue, presenting the rationale for encouraging the purchase of SPO consumables (20). These latter two interventions were both successful in increasing intentions to purchase SPO, but did not result in significant behavior change (20, 34). Therefore, although knowledge and awareness of palm oil and SPO has been shown to reliably predict SPO purchasing intentions and self-reported SPO purchasing behavior cross-sectionally (26), longer-term behavior change has not been observed following an increase in this knowledge (20, 34).

### Drivers and barriers of opportunity

Previous findings on green behavior initiatives have indicated that a focus on educational interventions is often insufficient to change behavior, thus requiring a greater focus on opportunity-related barriers (43). A factor that overlaps capability and opportunity is affordability, which has been identified as a significant obstacle to green purchasing behavior (12, 44, 45), alongside the expectation that sustainable products are more expensive (25). In the qualitative study that explored barriers to SPO purchasing using the COM-B framework, consumers acknowledged that irrespective of potential cost differences (i.e., between a product containing SPO versus uncertified palm oil), when financial pressures build up, shoppers are less likely to check labels for the ‘social welfare element’ (25). At the same time, other research has indicated that consumers are willing to pay a small premium for sustainably sourced certified palm oil, even in developing producer countries like Malaysia (46), particularly when provided with adequate information about the importance of sustainability with respect to palm oil (47). Additionally, consumers may also have limits on their time and energy levels with respect to grocery shopping (25). Although they may agree that SPO products are desirable, they may be unable to spend extra time and energy to scrutinize labels and seek SPO products. Additionally, it is worth noting that various black swan events (e.g., the COVID-19 pandemic) can drastically alter grocery shopping behavior (48), with people tending to shop faster than normal (49), prioritizing personal and family safety over ethical purchasing.

Another opportunity-related barrier is that consumers might underestimate the complexity of identifying SPO containing products (perceived product availability), including the time required for extensive research on which products contain SPO prior to the actual supermarket trip, as well as obscure and unclear product labels (25, 40). Previous research in the UK and Germany have indicated that very few consumers are aware of ecolabels with respect to certified SPO (47, 50), such as the Roundtable on Sustainable Palm Oil (RSPO) trademark or symbol (51). The RSPO is a non-governmental organization consisting of producer, trader, consumer, and investor stakeholders that was founded in 2004 to regulate and promote the use of SPO (52, 53). Despite mixed reviews about its success (9, 16, 54–56), the RSPO continues to revise its standards (53, 57), and the latest RSPO Impact Report shows promise (e.g., the total conservation areas under protection have more than doubled since 2015; the probability of a fire hotspot occurring within an RSPO certified plantation is less than 1.5%) (58). In addition to making them aware of the RSPO trademark, the online intervention research discussed earlier (20) presented participants with an Australian shopping guide (31) and a list of brands that consumers could target (32). However, these tools were unsuccessful in closing the intention-behavior gap for purchasing SPO products (20).

Closer analysis of the intervention study that had also presented information to increase awareness on SPO, revealed that perceived product availability significantly differentiated between those who engaged in SPO purchasing behavior, and those who expressed high intentions but did not engage in the behavior (20). Interestingly, the control group, who was not exposed to the educational intervention on palm oil and SPO, showed a non-significant trend toward better perceived product availability and increased follow-up SPO purchasing behavior (20). The authors postulated that the bid to increase knowledge and awareness of SPO might have highlighted the issue’s complexity, as well as the lack of an ideal solution, leading consumers to perceive increased difficulty in identifying and locating SPO products (20). There are many reasons why SPO products might be perceived as less available, including the use of tiny fonts in ingredient lists on product labels, an ecolabel that is not well-recognized (25), and the reluctance by manufacturers to use an ecolabel (or any other means to indicate the presence of SPO) due to negative public perceptions around palm oil in general (14, 38), even though research indicates that consumers may not actively seek to avoid ecolabels (50). Instead, palm oil (sustainable or otherwise) tends to be a hidden ingredient, referred to simply as “vegetable oil,” or under other technical terms, of which there are more than 200 (59) – all of which make it challenging for consumers to accurately identify palm oil products. None of the intervention research discussed above has been successful in addressing these specific opportunity-related barriers.

## Recommendations

For the time being palm oil is likely to remain a major player in the consumable oil market. It is more versatile and affordable than alternative oils (1) and substitute synthetic oils are years away from being financially practical (60). Currently, the best (albeit imperfect) solution at hand is to increase the demand and supply of SPO so that it replaces uncertified palm oil (1, 16, 60). This mini-review concludes with the finding that well-intentioned interventions to encourage consumers to purchase sustainably with respect to palm oil have had limited success, primarily owing to their failure to address significant opportunity-related barriers, particularly those related to poor product labeling. The following recommendations aim to address these barriers.

### Recommendation 1: altering policies on product labels

When national policies do not require palm oil to be labeled such (61), they can be easily subsumed under the generic ‘vegetable oil,’ or described by the almost 200 other terms denoting it (59). One policy recommendation would be to make it mandatory for producers to declare palm oil as an ingredient, and specify its certification status with respect to sustainability. Research has also indicated that consumers might respond more favorably to a label of ‘organic’ certified palm oil as a sustainability indicator (47), as this might be more familiar to them when compared to ‘sustainable.’

### Recommendation 2: addressing barriers to certification processes

It would also be beneficial for the RSPO to follow-up on their principles and criteria to ensure that all member organizations are committed to 100% SPO in products, with appropriate penalties in place if found slacking (55, 62). The RSPO also needs to address several valid criticisms, including a lack of accountability (being a private governing body, there is no external oversight), a complicated process required for certification (which makes it particularly difficult for small-scale farmers), costs (with references being made that the cost of certification far exceeds the selling price of SPO), and a potential lack of legitimacy in producer countries like Indonesia and Malaysia, which have their own certification systems in place (63). Further, small-scale farmers (also known as smallholders, who run 40% of the oil palm plantations; (1)) have limited understanding of the costs of RSPO certification, often require non-governmental organizational (NGO) support for certification, and perceive that the financial benefits of certification are small (64). Finally, membership in the RSPO is voluntary, and it may not be practical to ‘force’ producers to join. Therefore, the onus of responsibility currently lies with consumers, who are faced with the impossible task of identifying which producers are members of the RSPO, and which import 100% SPO (as versus mixed oils). With complex supply chains, there needs to be a certified chain of custody and transparency from producers and the RSPO that verifies them (63).

A qualitative single case study of a Malaysian palm oil company (that sources palm oil from plantations before selling them to companies) was conducted to understand what might be key drivers and/or barriers toward the move to 100% SPO from the producer’s perspective (65). The findings suggest that it is a chain reaction of pressure received from multiple external groups including NGOs, international buyers (big brands like Ferrero and Nestle), other competitors in the industry, investors, and financiers that strongly motivate the adoption of sustainability practices with respect to palm oil (65). This finding further strengthens the argument that we need to look beyond a singular focus on consumer purchasing power, and instead look to multiple sources of pressure for sustainable change.

### Recommendation 3: implementing national procurement policies

An overarching way forward might be the implementation of a national procurement policy like the one implemented in the UK in 2012, where they committed to sourcing only SPO by 2017 (66). While this target is yet to be achieved, it has set things in the right direction, with a definite increase in the country’s sourcing of SPO (66). If national law requires that producers/companies source only certified SPO, this will relieve consumers of the unfair responsibility of having to detect SPO-friendly brands themselves (50). This policy will also ensure that the burden of cost need not be borne by the consumer.

## Conclusion

In conclusion, attempts to close the intention-behavior gap for purchasing SPO products, thereby increasing the demand for such products, will be more successful if the opportunity-related barriers that consumers face are addressed by national policy changes, corporate responsibility, and increased accountability by the RSPO that make SPO affordable, identifiable, or even the norm. Experts have suggested that SPO use can be better increased if consumers pressure manufacturers and/or governments to source SPO for their products (16). Future studies should explore barriers that prevent consumers from engaging in these more effective activities and evaluate interventions designed to promote them. Furthermore, future research should move beyond the individual consumer to target the likely barriers to the implementation of our recommendations on a broader scale, specifically how to: (1) support the RSPO in enforcing their principles; (2) encourage manufacturers to increase transparency in labeling; and (3) motivate governments to implement SPO procurement policies.

## Author contributions

CS: Conceptualization, Writing – original draft, Writing – review & editing. AL: Writing – review & editing. DH: Writing – review & editing.
